# Targeting HER3, a Catalytically Defective Receptor Tyrosine Kinase, Prevents Resistance of Lung Cancer to a Third-Generation EGFR Kinase Inhibitor

**DOI:** 10.3390/cancers12092394

**Published:** 2020-08-24

**Authors:** Donatella Romaniello, Ilaria Marrocco, Nishanth Belugali Nataraj, Irene Ferrer, Diana Drago-Garcia, Itay Vaknin, Roni Oren, Moshit Lindzen, Soma Ghosh, Matthew Kreitman, Jeanette Clarissa Kittel, Nadege Gaborit, Gretchen Bergado Baez, Belinda Sanchez, Raya Eilam, Eli Pikarsky, Luis Paz-Ares, Yosef Yarden

**Affiliations:** 1 Department of Biological Regulation, Weizmann Institute of Science, Rehovot 76100, Israel; romaniellodonatella@gmail.com (D.R.); ilaria.marrocco@weizmann.ac.il (I.M.); nishanth.nataraj@weizmann.ac.il (N.B.N.); diana.drago@weizmann.ac.il (D.D.-G.); itay.vaknin@weizmann.ac.il (I.V.); moshit.lindzen@weizmann.ac.il (M.L.); soma.ghosh@weizmann.ac.il (S.G.); matthew.kreitman@weizmann.ac.il (M.K.); jc.kittel@web.de (J.C.K.); 2 Centro de Investigación Biomédica en Red de Cáncer (CIBERONC), 28029 Madrid, Spain; ireneferrersan@gmail.com (I.F.); lpazaresr@seom.org (L.P.-A.); 3 Lung Cancer Clinical Research Unit, Instituto de Investigación Hospital 12 de Octubre & Centro Nacional de Investigaciones Oncológicas (CNIO), 28029 Madrid, Spain; 4 Department of Veterinary Resources, Weizmann Institute of Science, Rehovot 76100, Israel; roni.oren@weizmann.ac.il (R.O.); raya.eilam@gmail.com (R.E.); 5 Institut de Recherche en Cancérologie de Montpellier (IRCM), INSERM U1194, Université de Montpellier, 34298 Montpellier, France; nadege.gaborit@igh.cnrs.fr; 6 Institut Régional du Cancer de Montpellier (ICM), 34298 Montpellier, France; 7 Tumor Biology Direction, Center of Molecular Immunology, Havana 11600, Cuba; gretchen@cim.sld.cu (G.B.B.); belinda@cim.sld.cu (B.S.); 8 The Lautenberg Center for Immunology and Cancer Research, Faculty of Medicine, Hebrew University of Jerusalem, Jerusalem 91120, Israel; peli@hadassah.org.il; 9 Medical Oncology Department, Hospital Universitario 12 de Octubre, 28041 Madrid, Spain

**Keywords:** drug resistance, EGFR, kinase inhibitor, lung cancer, monoclonal antibody, osimertinib

## Abstract

Although two growth factor receptors, EGFR and HER2, are amongst the best targets for cancer treatment, no agents targeting HER3, their kinase-defective family member, have so far been approved. Because emergence of resistance of lung tumors to EGFR kinase inhibitors (EGFRi) associates with compensatory up-regulation of HER3 and several secreted forms, we anticipated that blocking HER3 would prevent resistance. As demonstrated herein, a neutralizing anti-HER3 antibody we generated can clear HER3 from the cell surface, as well as reduce HER3 cleavage by ADAM10, a surface metalloproteinase. When combined with a kinase inhibitor and an anti-EGFR antibody, the antibody completely blocked patient-derived xenograft models that acquired resistance to EGFRi. We found that the underlying mechanism involves posttranslational downregulation of HER3, suppression of MET and AXL upregulation, as well as concomitant inhibition of AKT signaling and upregulation of BIM, which mediates apoptosis. Thus, although HER3 is nearly devoid of kinase activity, it can still serve as an effective drug target in the context of acquired resistance. Because this study simulated in animals the situation of patients who develop resistance to EGFRi and remain with no obvious treatment options, the observations presented herein may warrant clinical testing.

## 1. Introduction

Members of the human epidermal growth factor receptor (EGFR/HER) family, which also includes, in addition to EGFR, HER2, HER3, and HER4, have been found overexpressed or mutated in several types of cancer [1]. For instance, 10–30% of all patients with NSCLC (non-small cell lung cancer) carry activating mutations in the gene encoding EGFR [2,3,4,5,6]. The most common mutations are an exon 19 short deletion (E746–A750) and an exon 21 point mutation, L858R [7]. Tumors harboring EGFR mutations are often treated with tyrosine kinase inhibitors (TKIs) like gefinitib and erlotinib, which belong to the first generation of clinically approved EGFRi [8,9,10]. Despite initial efficacy, patients eventually become resistant to these drugs. The most common mechanism of resistance entails emergence of a secondary mutation (T790M) [11]. Other major mechanisms include amplification of genes encoding other receptor tyrosine kinases (RTKs), especially *MET* [12] and *HER2* [13], overexpression of the hepatocyte growth factor, the respective receptor, MET [14,15], or yet another RTK, AXL [16].

Trials that compared osimertinib, a third-generation TKI, to chemotherapy in drug-resistant T790M-positive patients demonstrated that osimertinib achieved superior progression-free survival over chemotherapy [17], which led to the approval of osimertinib in 2015. More recently, osimertinib has been approved also as a first-line treatment [18]. Despite impressive therapeutic effects, secondary resistance to osimertinib is an emerging issue. The most common mechanisms of resistance to osimertinib in first-line settings are *MET* or *HER2* amplification, the C797S mutation, and mutations in downstream signaling proteins [19]. In general, treatment with TKIs generates a selective pressure to circumvent the inhibited kinase through ‘bypass tracks’ [20,21]. Accordingly, amplification of *MET*, which occurs in approximately 5% of tumors with acquired refractoriness to EGFR inhibitors, confers resistance by driving HER3-mediated activation of downstream PI3K-AKT signaling [12]. In addition, HER3 might assist emergence of resistance due to a compensatory shift in the HER3 phosphorylation-dephosphorylation equilibrium [22]. Mechanistically, HER3 acts as the preferred heterodimerization partner of EGFR and HER2, such that upregulation of this receptor can sustain several survival pathways [11,23]. In line with these observations, neuregulin 1 (NRG1), a ligand of HER3, is the strongest mitogenic factor for NSCLC cells [24]. Furthermore, upregulation of the NRG1-HER3 axis can mediate escape from various anti-HER therapies [25]. Apparently, a PI3K- and FoxO3a-dependent up-regulation of HER3 limits the antitumor action of TKIs targeting ERBB/HER family members [26].

In similarity to the well-established involvement of HER3, *HER2* amplification has been detected in 12% of lung tumors with acquired resistance to EGFR therapy [13]. These observations raised the possibility that co-targeting EGFR, along with HER3 or HER2, might delay resistance to TKIs. Accordingly, we previously treated erlotinib-resistant (EGFR-T790M) models with a combination of osimertinib and three antibodies targeting EGFR, HER2, and HER3 [27,28]. Short treatments that combined all four drugs cured an erlotinib-resistant xenograft model. In an effort to reduce the number of drugs, our next studies combined osimertinib with two clinically approved monoclonal antibodies (mAbs), cetuximab (an anti-EGFR mAb) and trastuzumab, an anti-HER2 antibody. These studies revealed that continuous schedules of concurrent treatments were essential for inhibition of cell line xenografts [29]. Herein we show, for the first time, that the efficacy of the triple drug combination (i.e., osimertinib, cetuximab, and trastuzumab) extends to genetically more heterogeneous xenografts, namely patient-derived xenografts (PDX). In addition, we present evidence in favor of co-targeting the other partner of EGFR, HER3, as a strategy suitable for long-term delay of secondary resistance. We report that the mechanism underlying the effects of co-targeting HER3 entails prevention of TKI-induced upregulation of this kinase-defective receptor, suppression of MET activation and AXL upregulation, along with inhibition of AKT signaling and upregulation of a pro-apoptosis protein. Taken together, the observations we made in animals and in vitro offer a novel therapeutic strategy capable of preventing resistance to the latest generation EGFR-specific TKIs.

## 2. Results

### 2.1. Combining Osimertinib with Antibodies against EGFR and HER2 Overcomes Resistance of PDX Models

**Figure 1:**
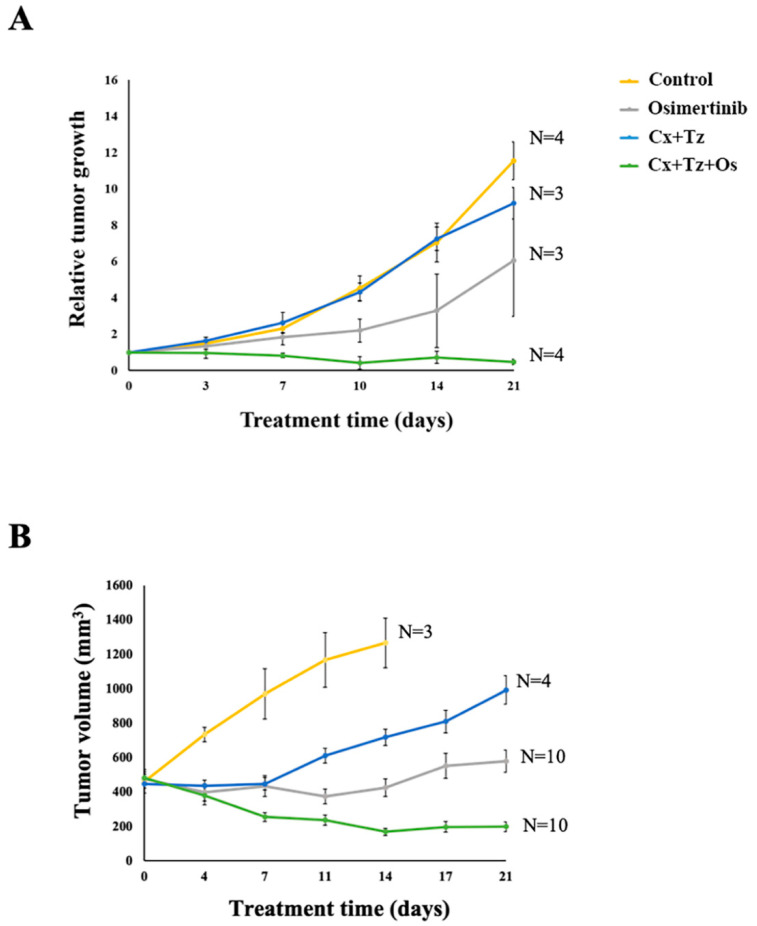
Combining osimertinib and mAbs neutralizing EGFR and HER2 strongly inhibits two EGFR^+^ patient-derived xenograft models of non-small cell lung cancer (NSCLC). (**A**) The lung TP103 patient-derived xenograft (PDX) model (harboring two EGFR mutations: L858R and T790M) was implanted in the flanks of CD1-nu/nu mice. Animals with palpable tumors were divided in four groups: Vehicle control, daily osimertinib (Os, 5 mg/kg/mouse), a combination of two antibodies, cetuximab (Cx), and trastuzumab (Tz, intraperitoneally injected with 0.1 mg of each mAb, per mouse). An additional group was treated with a mixture of the antibodies and osimertinib. Treatment was terminated after 21 days. Tumor volumes and body weights were monitored twice and once a week, respectively. Data shown are the means ± SEM. The number (N) of mice per group is indicated. (**B**) The lung TM00219 (PDXJ2) PDX model, which was derived from a metastatic tumor carrying two EGFR mutations (delE746–A750 and T790M) was employed. Tumors were engrafted and expanded in NSG mice. Mice were treated as in A.

The efficacy of combining a TKI and two mAbs, to EGFR and HER2, has so far been determined with cell lines cultivated in monolayer and xenografts derived from them [29]. These models are unlikely to represent the genetic and clonal heterogeneity of NSCLC. In contrast, patient-derived xenograft (PDX) models, based on implantation of cancer tissue specimens from patients into immunodeficient mice, are more reliable [30]. To test the efficacy of combining osimertinib and antibodies neutralizing EGFR and HER2 (cetuximab, Cx, and trastuzumab, Tz, respectively), we made use of two PDX models: The lung TP103 model (harboring two EGFR mutations: L858R and T790M) and the TM00219 (PDXJ2) model, which was derived from a metastatic tumor carrying delE746–A750 (along with T790M). Animals with palpable tumors were divided in four groups: Vehicle control, daily osimertinib (Os, 5 mg/kg/mouse), or a combination of two antibodies, cetuximab and trastuzumab (each mAb at 0.1 mg, per injection). An additional group was treated with a mixture of the antibodies and osimertinib. Although different mutations drive the two models, the results we observed were quite similar (Figure 1): Each untreated cohort displayed rapid tumor growth, which was either unaffected or partly inhibited by the combination of two mAbs. Although osimertinib retarded the rates of growth of both models, no tumor shrinkage occurred. As expected, both models were strongly inhibited by the combination of osimertinib and the antibodies, and each model exhibited clear regression after treatment. These results underscore the cooperative features of the drug combination we employed.

### 2.2. An Anti-HER3 Antibody Prevents Osimertinib-Induced Upregulation of HER3, as well as Inhibits Release of the Soluble Extracellular Domain

**Figure 2:**
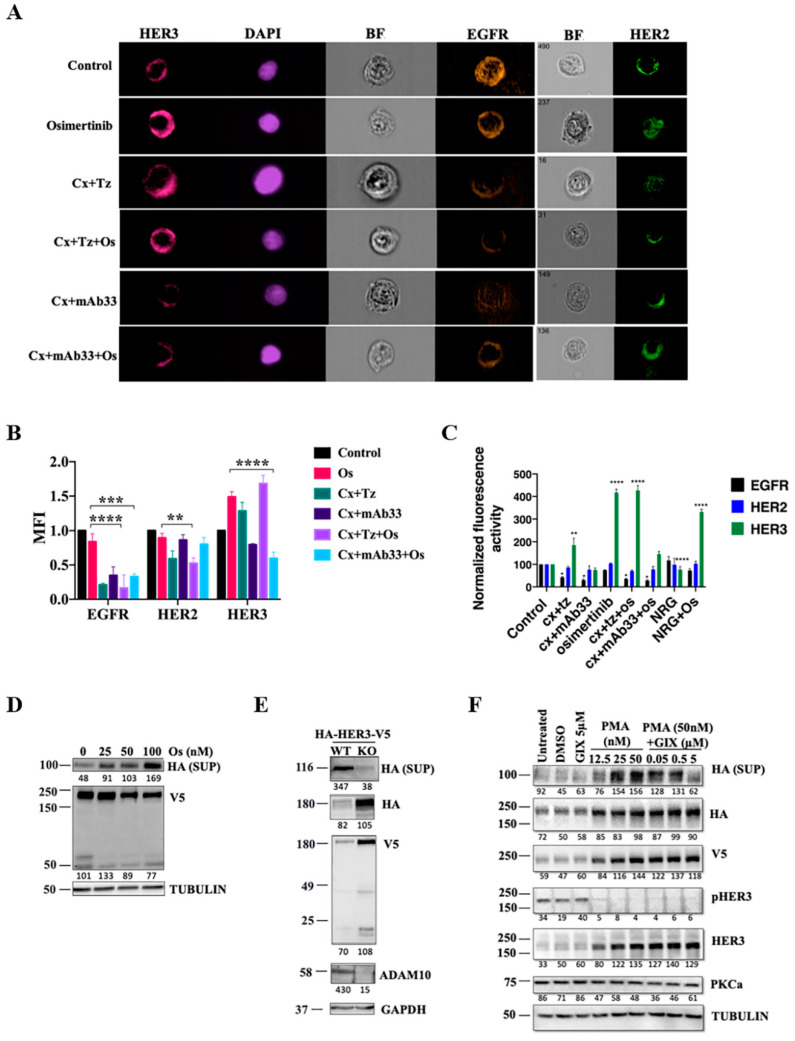
Osimertinib enhances cleavage of HER3 and induces up-regulation of the kinase-dead receptor, but a HER3-neutralizing antibody prevents up-regulation. (**A**) PC9ER cells were treated with osimertinib (40 nM), or with the indicated pairs of antibodies (each at 0.01 mg/mL; total antibody concentration: 0.02 mg/mL), either alone or in combination with osimertinib. After 24 h, the cells were washed in acidic buffer and harvested. Permeabilization was carried out by adding 0.05% Triton X-100 followed by 1 h of blocking. Thereafter, cells were stained with the corresponding primary antibody. DAPI was used to stain nuclei. After several washes, samples were analyzed using the ImageStream^®^X Mk II Imaging Flow Cytometer. BF, bright field. (**B**) Signals obtained from 50,000 cells analyzed as in A were integrated, normalized, and presented. The results shown represent the average of two independent experiments. Values represents averages ± SEM. Significance was assessed using one-way ANOVA and the Dunnett’s multiple comparison test. **** *p* < 0.0001, *** *p* < 0.001, ** *p* < 0.01. (**C**) PC9ER cells (5 × 10^5^) were incubated in media containing serum (1%) and vehicle (DMSO or saline), osimertinib (40 nM), or the indicated pairs of cetuximab (Cx), trastuzumab (Tz) and an anti-HER3 antibody (mAb33; each at 0.01 mg/mL). Flow cytometry was performed to analyze cell surface expression of EGFR, HER2, and HER3. The median fluorescence intensity of two independent experiments is shown. Data were normalized to the control. Significance was assessed using two-way ANOVA with Tukey’s multiple comparison test. (**D**) PC9ER cells were transfected with a plasmid encoding HER3 tagged with HA (N-terminus) and V5 (C-terminus) peptides. Thereafter, cells were starved overnight, and treated for 24 h with vehicle (DMSO) or with increasing concentrations of osimertinib. Supernatants (SUP) and whole cell lysates were harvested and immunoblotted. Note that soluble HER3 was detected in supernatants by using antibodies against HA, while the full-length form and the intracellular domain of HER3 were detected by using antibodies against V5. (**E**) Wild type (WT) and ADAM10^-/-^ (KO) mouse embryo fibroblasts (MEFs) were transfected with the HA-HER3-V5 plasmid. After 48 h, supernatants and lysates were harvested and immunoblotted with the indicated antibodies. (**F**) PC9ER cells stably expressing the HA-HER3-V5 construct were treated for 6 h with the indicated concentrations of phorbol myristate acetate (PMA) in the absence or presence of GI254023 (GIX). After 6 h, supernatants and whole cell lysates were harvested and immunoblotted with the indicated antibodies. Signals were quantified and normalized (numbers shown below each lane). GAPDH or TUBULIN were used as loading control. The locations of molecular weight markers are indicated.

Combining drugs like osimertinib, cetuximab, and trastuzumab might translate to increased toxicity. Especially worrisome is the use of trastuzumab, since targeting this co-receptor might cause severe heart failures due to myocyte dysfunction [31]. Herein, we addressed the possibility that targeting HER3 can offer a therapeutic alternative. To begin with, we followed changes in HER3 abundance in erlotinib-resistant PC9ER lung cancer cells (delE746–A750 plus T790M) pre-exposed to combinations of osimertinib, cetuximab, trastuzumab, and mAb33, a neutralizing anti-HER3 mAb we previously generated [32]. Immunofluorescence analysis confirmed that cetuximab and trastuzumab downregulated the respective target receptors, whereas osimertinib strongly up-regulated HER3 and weakly increased HER2 and EGFR (Figure S1A). Importantly, combining osimertinib with cetuximab and mAb33 prevented TKI-induced up-regulation of HER3. This effect, we assume, is due to the ability of mAb33 to stimulate endocytosis and intracellular degradation of HER3. To verify these effects, we employed the ImageStream^®X^ Mk II Imaging Flow Cytometer (EMD Amnis, Seattle, WA, USA). By collecting 50,000 digital images per sample and providing a numerical representation of image-based features (Figure 2A,B), we concluded that osimertinib specifically elevated HER3 abundance. Consistent with Figure S1A, cetuximab and trastuzumab downregulated the respective receptors and mAb33 blocked the ability of osimertinib to up-regulate HER3. Next, we measured surface receptor levels by applying conventional flow cytometry (Figure 2C). The results confirmed the inferred ability of osimertinib to elevate surface HER3 (>4-fold), but this was almost nullified when mAb33 was co-administered. To resolve the mechanism underlying osimertinib-induced upregulation of HER3, we isolated mRNAs from drug-treated PC9ER and H1975 (EGFR L858R and T790M) cells. PCR analysis revealed that the TKI strongly elevated transcripts encoding HER3, as well as HER2 (Figure S1B). These results indicated that the effect of osimertinib on HER3 was primarily at the level of transcription, whereas the effect of mAb33 was posttranslational.

A naturally occurring secreted form of HER3 (sHER3) is a potent negative regulator of NRG [33], and plasma levels of sHER3 might predict survival of patients with bladder cancer [34]. To address potential TKI-induced up-regulation of sHER3, we made use of a dual peptide-tagged HER3. Analysis of sHER3, using antibodies specific to the N-terminal tag (hemagglutinin A; HA) detected osimertinib-induced up-regulation of sHER3 (Figure 2D). Reciprocally, using antibodies specific to the carboxyl-terminal tag confirmed parallel depletion of full-length HER3. Notably, several RTKs serve as substrates of two members of the disintegrin and metalloproteinase domain-containing family, ADAM10 and ADAM17 [35]. Hence, we employed a line of fibroblasts that expresses no ADAM10. Cleavage of HER3 in this cell line was strongly, but incompletely, reduced (Figure 2E). Congruently, cleavage was inhibited by GI-254023X, an ADAM10 inhibitor. The residual ADAM10-independent cleavage might be executed by ADAM17, because phorbol myristate acetate (PMA), which specifically stimulates ADAM17 [36], enhanced sHER3 (Figure 2F). Consistent with TKI-induced activation of ADAM10, we detected high sHER3 in medium conditioned by osimertinib- or PMA-treated PC9ER cells, but mAb33 did not affect HER3 cleavage (Figure S2A). In conclusion, osimertinib up-regulates transcription of *HER3*, as well as elevates ADAM-mediated cleavage. When this TKI was combined with pairs of mAbs, cetuximab+anti-HER3 or cetuximab + trastuzumab, the former combination uniquely prevented HER3 up-regulation.

### 2.3. In Combination with Osimertinib and Cetuximab, the Anti-HER3 mAb Enhances Apoptosis

**Figure 3:**
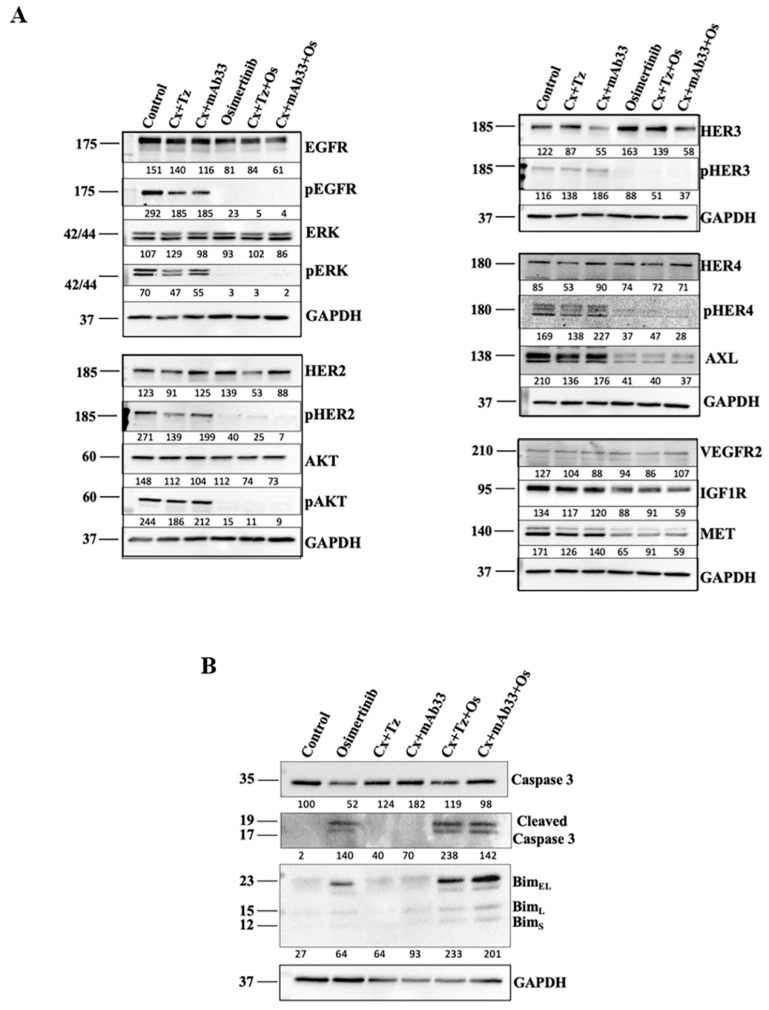
Combining an anti-HER3 mAb with osimertinib and cetuximab downregulates HER3, EGFR, and other RTKs and increases BIM. PC9ER cells were treated for 24 h with osimertinib (40 nM), the indicated pairs of antibodies (0.01 mg/mL each), and the respective drug combinations. Proteins were extracted, blotted, and probed for the indicated markers of signaling (**A**) or apoptosis (**B**). GAPDH was used as a marker of equal protein loading. Signals were quantified and normalized (numbers shown below each lane).

To validate the ability of mAb33 to downregulate HER3, a molecule that can translocate to the nucleus [37], we applied immunoblotting (Figure 3A). PC9ER cells pre-treated for 24 h with mAb33, in combination with either cetuximab or cetuximab+osimertinib, displayed no effect on HER2 but markedly reduced HER3. Importantly, the latter treatment, in similarity to osimertinib alone, destabilized two other resistance associated RTKs, namely AXL and MET, as well as erased the active forms of EGFR, HER2, HER3, and HER4. These effects were shared by the other triplet, osimertinib + cetuximab + trastuzumab, which downregulated HER2, rather than HER3, and they were accompanied by complete inactivation of AKT and ERK (Figure 3A and Figure S2B). Notably, the most prominent difference between the triplets, as compared to osimertinib only, was downregulation of the target receptor, HER3 in the case of mAb33. Hence, we asked if these differences translate to effects on cell death markers. Two markers, cleaved caspase 3 and BIM, were moderately induced by osimertinib, but further enhanced after treatment with either combination of osimertinib + 2XmAbs (Figure 3B). Of relevance, expression levels of BIM can predict response and duration of clinical benefit from EGFR inhibitors [38]. Conceivably, by removing multiple RTKs from the cell surface, the combination of mAb33, cetuximab, and osimertinib blocks critical survival pathways.

**Figure 4:**
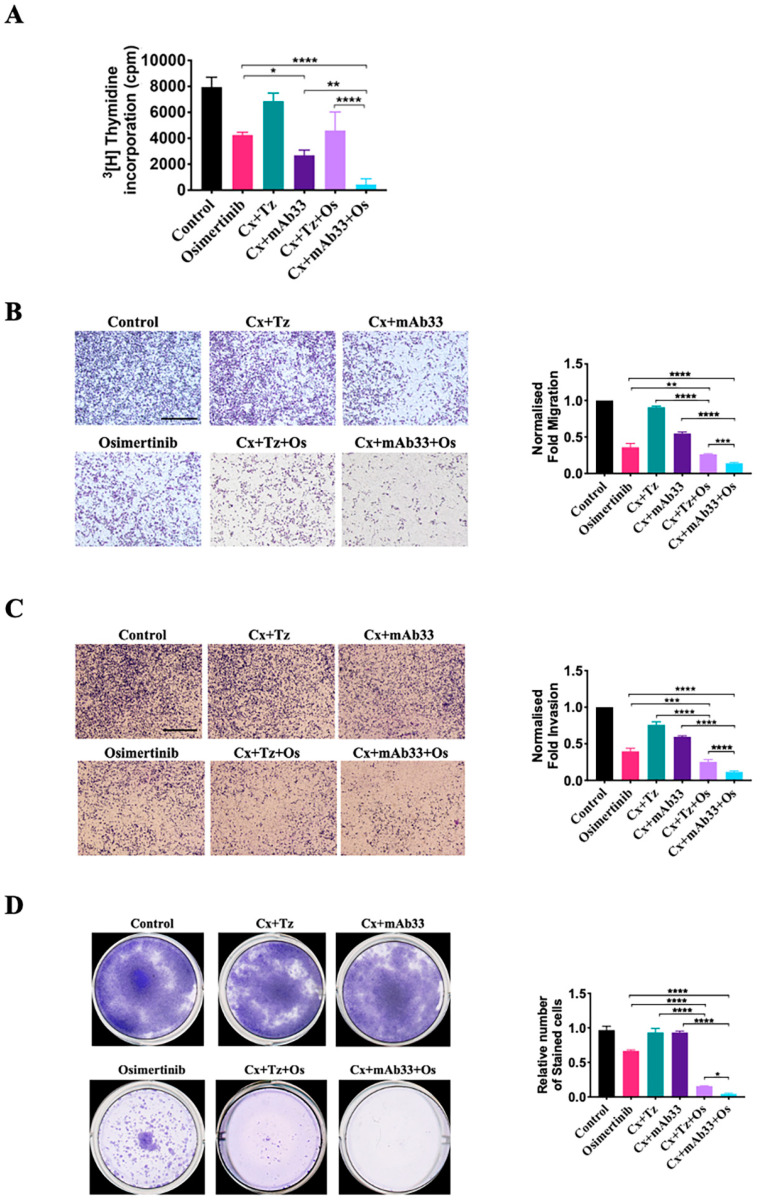
Assays of DNA synthesis, cell motility and clonogenicity indicate superiority of an anti-HER3 mAb over an anti-HER2 antibody. (**A**) Thymidine incorporation assays were performed with PC9ER cells pre-plated onto 24-well plates at a density of 2×10^4^ cells/well. After 12 h the medium was replaced with fresh serum-free medium containing radioactive thymidine and drugs. The following treatments were applied: cetuximab + trastuzumab, cetuximab + mAb33 (each antibody was applied at 0.01 mg/mL), osimertinib (40 nM), and the respective combinations. The incorporation of radioactive thymidine into DNA was assayed 48 h later in quadruplicates and repeated twice. Significance was assessed using two-way ANOVA followed by Sidak’s multiple comparison test. Values represent the means ± SEM. Cell migration (**B**) and cell invasion (**C**) assays were performed with PC9ER cells that were subjected to the following treatments for 48 h: The indicated pairs of mAbs (0.01 mg/mL each), osimertinib (40 nM), and the respective combinations of osimertinib and a pair of mAbs. Pretreated cells were plated in triplicates in transwells or in invasion compartments. Migration and invasion were carried out for 20 and 22 h, respectively, in complete medium. Shown are representative images and the respective histograms. Signals were normalized to control. Significance was assessed using one-way ANOVA followed by Dunnett’s or Tukey’s multiple comparison test. Values represents averages ± SEM. Each experiment was repeated thrice. Bars, 100 µm. (**D**) Colony outgrowth assays were performed with PC9ER cells (1 × 10^3^) that were seeded in 12-well microplates. Cells were later exposed to the following drugs for 9–10 days: Osimertinib (40 nM), the indicated pairs of two mAbs (each at 0.01 mg/mL), and their combination with osimertinib. Thereafter, cells were fixed in formaldehyde (4%) and stained with crystal violet (0.5%). Photos were taken using an EPSON scanner. Cell growth was quantified by dissolving crystal violet in detergent (2% SDS). Light absorbance was quantified at 590 nm. The experiment was repeated thrice. Significance was assessed using one-way ANOVA with Tukey’s multiple comparison test. **** *p* < 0.0001, *** *p* < 0.001, ** *p* < 0.01, * *p* < 0.05.

Interestingly, trastuzumab stimulated, in some assays, tyrosine phosphorylation of HER2, especially following prolonged incubations (Figure S2B). This may be due to agonistic activity of the bivalent mAb, but no similar activity could be attributed to mAb33, probably because of the impaired catalytic function of HER3. To unravel additional features that might differentiate between the two treatments, we applied a series of assays on PC9ER cells (EGFR delE746–A750 and T790M; Figure 4) and H1975 cells (EGFR L858R and T790M; Figure S3). Firstly, we assayed the incorporation of radioactive thymidine into DNA of dividing cells (Figure 4A and Figure S3A). While osimertinib and mAb pairs were partly inhibitory, co-targeting HER3 and EGFR was far more potent than targeting EGFR and HER2, and the triplet cetuximab + mAb33 + osimertinib nearly blocked DNA synthesis. Similar conclusions were reached when we applied two cell motility assays. The first, a cell migration assay, utilized inserts nested in wells of culture plates (Figure 4B and Figure S3B). The second test, a cell invasion assay, measured the capacity to penetrate through an extracellular barrier (Figure 4C and Figure S3C). Notably, the results from both cell lines confirmed that co-targeting HER3 was more efficacious than co-blocking HER2.

The fourth test, a colony forming assay, assessed the capacity to generate colonies of 50 or more cells. Initially, PC9ER (Figure 4D) and H1975 (Figure S3D) cells were seeded in microplates and later exposed to the drugs for 9–10 days. The results confirmed strong cooperative effects: When singly used, the TKI and the pairs of mAbs induced relatively small effects, however both drug triplets almost completely blocked colony formation. In summary, the results we obtained portrayed a consistent picture: Osimertinib collaborated with both mAb pairs, but the pair containing mAb33 appeared more potent in all four in vitro assays we performed.

### 2.4. By Inhibiting HER3 and Several Other RTKs, a Combination of an Anti-HER3 mAb, Cetuximab, and Osimertinib Induces Complete Regression of a Cell Line Xenograft Model

**Figure 5:**
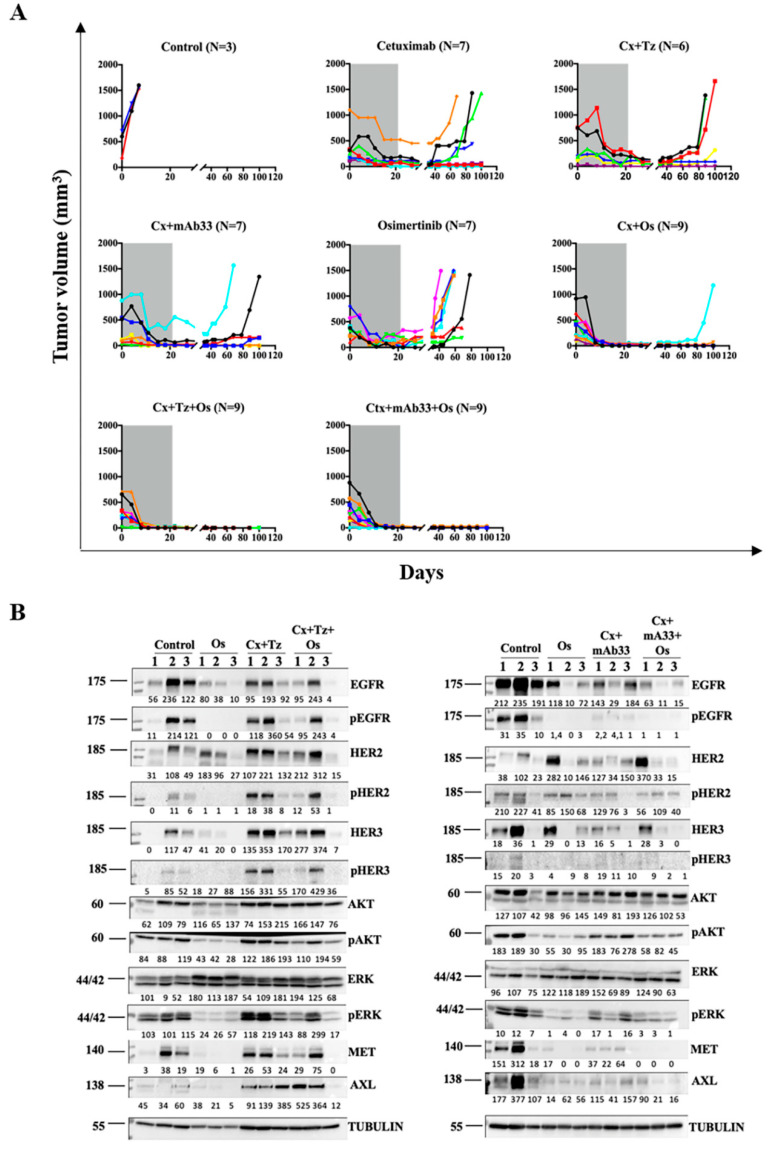
An anti-HER3 mAb, in combination with cetuximab and osimertinib, persistently eradicates erlotinib-resistant tumors and eliminates several RTKs in a xenograft model. (**A**) CD1 nu/nu mice were injected with PC9ER cells (E746–A750 deletion and the T790M mutation; 3 × 10^6^ cells per mouse). Mice harboring palpable tumors were divided into eight different treatment groups: (i) Vehicle control, (ii) daily oral gavage of osimertinib (administered at 5 mg/kg/mouse as a single drug, and 1 mg/kg/mouse when applied in combination with mAbs), (iii) cetuximab (0.2 mg/injection/mouse), (iv) cetuximab plus osimertinib, (v and vi) the following mAb pairs: Cetuximab plus trastuzumab and cetuximab plus mAb33 (each mAb at 0.1 mg per injection), and (vii and viii) combinations comprising osimertinib and the respective pairs of mAbs. The antibodies were intraperitoneally injected twice a week. Treatments continued until day 21 and tumor volume was monitored for the next four months. Mice were euthanized when tumor size reached 1500 mm^3^. (**B**) An in vivo experiment was performed with three mice per group, essentially as in A. Mice were sacrificed after seven days of treatment. The respective tumors were extracted, and the extracts processed for immunoblotting that used the indicated antibodies.

The cooperative drug–drug interactions we observed in vitro prompted tests that used xenografts. The first trial entailed treatment of pre-established cell line xenografts. CD1 nu/nu mice carrying palpable PC9ER tumors were randomized into eight treatment groups (Figure 5A): (i) Vehicle control, (ii) osimertinib, (iii) cetuximab, (iv) cetuximab plus osimertinib, (v) cetuximab plus trastuzumab, (vi) cetuximab plus mAb33, (vii) osimertinib plus cetuximab plus trastuzumab, and (viii) osimertinib plus cetuximab plus mAb33. The antibodies were delivered twice a week, for three weeks, and relapses were followed for up to 12 additional weeks. This protocol was selected because long drug holidays can uncover differences in drug potency and ability to control residual disease. As expected, both monotherapies we applied, osimertinib and cetuximab, caused rapid, yet incomplete tumor shrinkage, followed by recurrence during the drug holiday. Similarly, none of the three pairs of drugs, cetuximab + trastuzumab, cetuximab + mAb33, and cetuximab + osimertinib, fully prevented relapses, but the latter pair was clearly superior. In contrast, both drug triplets were curative: Within 2–3 weeks all tumors disappeared, and none relapsed after we stopped all treatments. Notably, although body weight of all mice was measured once a week, we observed no significant deviation from the control group (Figure S6A), indicating absence of overt toxic effects.

To help resolve the molecular basis, we re-performed the animal trial, but mice were sacrificed shortly after treatment onset (on day 7). Thereafter, tumors were processed for immunoblotting. Despite inter-animal variation, the results linked the therapeutic effects to the ability of mAb33 to downregulate not only HER3, but also AXL and MET. Thus, in at least two of three mice, treatment with the mAb33-containing triplet nearly erased EGFR, HER2, HER3, MET, and AXL (Figure 5B). Consequently, the active forms of both AKT and ERK were severely reduced. Slightly weaker effects were observed post treatment with the other triplet of drugs (osimertinib + cetuximab + trastuzumab). In conclusion, when combined with cetuximab and osimertinib, the anti-HER3 mAb we used prevented relapses of xenografts long after all treatments were stopped, and this might be attributed to simultaneous inactivation of several resistance-conferring RTKs.

### 2.5. In Combination with Osimertinib and Cetuximab, the Anti-HER3 mAb Inhibits a PDX Model and Delays Post-Treatment Relapses

**Figure 6:**
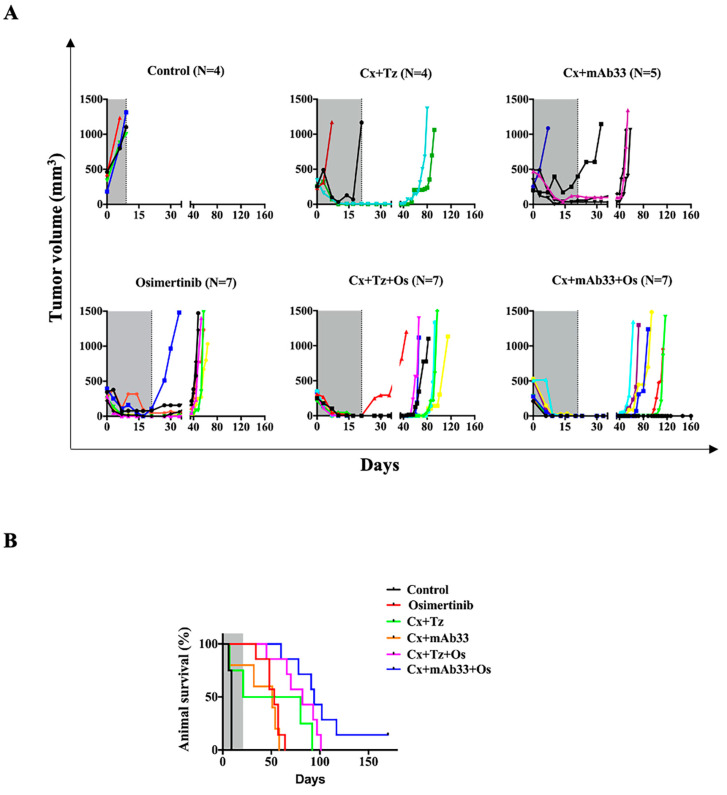
When applied on a model derived from a patient’s primary tumor, an anti-HER3 mAb, in combination with osimertinib and cetuximab, inhibits pre-established tumors and delays post-treatment relapses. (**A**) The lung PDX model TM00204 (PDXJ1), which was derived from a primary tumor (EGFR delE746–A750 and T790M), was engrafted and expanded in NSG mice that were treated as follows: Control, osimertinib (daily oral gavage, 10 mg/kg), combinations of antibody pairs (each at 0.1 mg/injection), either cetuximab plus trastuzumab (intraperitoneal delivery, twice per week), or cetuximab plus mAb33. Two additional groups of animals were similarly treated with osimertinib in combination with the indicated pairs of mAbs. All treatments were stopped after 21 days, but tumor growth was monitored up to day 160. Mice were euthanized when tumor size reached 1200 mm^3^. (**B**) Shown are survival plots corresponding to the groups of animals shown in A.

**Figure 7:**
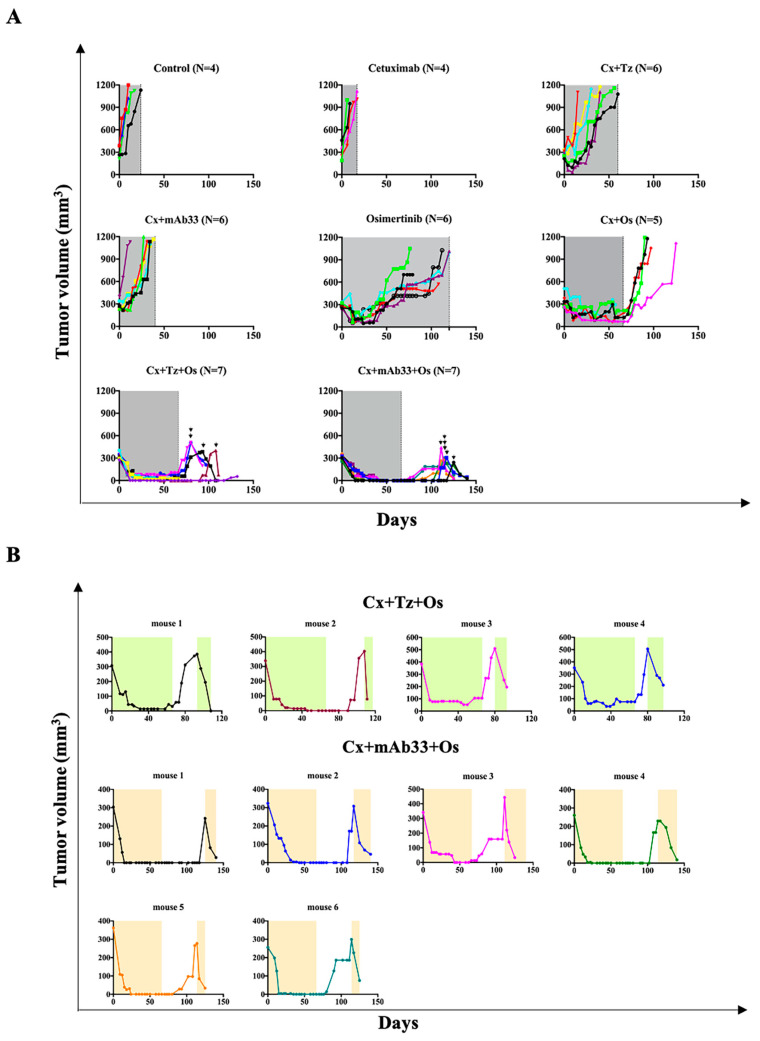
When applied on a model derived from a patient’s metastatic lesion, a drug combination containing an anti-HER3 mAb strongly inhibits pre-established tumors, delays post-treatment re-growth, and effectively inhibits residual disease. (**A**) The lung PDX model TM00219 (PDXJ2; derived from a metastatic lesion; EGFR delE746–A750 plus T790M) was engrafted in NSG mice, and later divided into eight groups, as follows: (i) Control, (ii) osimertinib (10 mg/kg/mouse), (iii) cetuximab alone (0.2 mg per injection; mice were treated twice weekly), (iv) cetuximab plus osimertinib, (v and vi) a pair of antibodies, cetuximab, and trastuzumab, or cetuximab and mAb33 (each at 0.1 mg per injection), and (vii and viii) two groups that were treated for 66 days with a combination of osimertinib (10 mg/kg/mouse) and a pair of antibodies. Animals were monitored until tumors relapsed. Relapsing tumors from the last two groups were re-treated (vertical arrowheads; see panel B). (**B**) Shown are tumor growth curves; each panel corresponds to a single animal, which was pre-treated for 66 days with a combination of osimertinib and a pair of mAbs. Note that two out of seven mice belonging to the group treated with cetuximab + trastuzumab + osimertinib died in the course of treatment (due to reasons unrelated to the tumors) and a third mouse died on day 125. Likewise, one mouse belonging to the group treated with cetuximab + mAb33 + osimertinib died in the course of treatment. Pre-treatments and re-treatments used the same combination of drugs. Colored areas indicate treatment phases and uncolored areas represent drug holidays.

Intra-tumor heterogeneity (ITH) might influence tumor aggressiveness, immunity, and response to therapy. To examine the ability of anti-HER3 antibodies to augment the combined action of osimertinib and cetuximab in the context of high ITH tumors, we employed two different PDX models, one derived from a primary tumor (Figure 6) and the other from a metastatic lesion (Figure 7). Note that due to logistics reasons, the numbers of experimental arms and mice per arm were limited. Animals bearing established tumors (>250 mm^3^) of the first model, TM00204 (PDXJ1; EGFR delE746–A750 and T790M), were randomized to the following groups: One group received osimertinib (10 mg/kg), two groups received antibody pairs (cetuximab + trastuzumab or cetuximab + mAb33), and two additional groups were treated with osimertinib in combination with a distinct pair of mAbs. All treatments were stopped after 21 days, but tumor growth was monitored up to day 160 and body weight was measured once a week (Figure 6A). Although osimertinib partly inhibited tumor growth, all tumors relapsed within 2–3 weeks after treatment cessation. Weaker effects were induced by the two pairs of mAbs. In contrast, both triple combinations completely and rapidly regressed pre-established tumors, as well as delayed the onset of relapses for longer intervals than the other treatments. Eventually, however, six of seven mice treated with cetuximab + mAb33 + osimertinib experienced relapses, while all seven mice treated with cetuximab + trastuzumab + osimertinib displayed recurrence. Importantly, we observed no signs of distress and monitoring body weights confirmed absence of marked toxicity (Figure S6B). Figure 6B presents the respective animal survival curves. Notably, no significant toxic effects were observed in terms of animal movements, body posture, and fur appearance. In summary, despite high ITH and aggressiveness of the PDX model we employed, the triplet utilizing an anti-HER3 mAb, similar to the trastuzumab-containing triplet, completely regressed pre-formed tumors, prevented relapses, as long as treatment continued, and delayed relapses after treatment cessation.

### 2.6. The Drug Combination Comprising an Anti-HER3 mAb Delays Relapse of a Model Derived from a Patient’s Metastatic Lesion

The other PDX model we employed, TM00219 (PDXJ2; derived from a metastatic lesion; EGFR delE746–A750 plus T790M), was engrafted in NSG mice, which were later divided into eight groups. Due to high aggressiveness, we extended treatment of pre-formed tumors to 66 days (120 days in the case of the osimertinib-treated group), unless relapses occurred earlier. Thereafter, animals were monitored until tumors relapsed. In similarity to the other PDX model, mice treated with osimertinib initially responded but later relapsed, while still under treatment (Figure 7A). Although the addition of cetuximab improved the outcome achieved by osimertinib alone, while inducing distress (see body weight monitoring in Figure S6C), the best results were observed in the groups of mice treated with the triplets, especially the one comprising mAb33: All tumors regressed and no relapses occurred as long as treatment continued. Nevertheless, both groups eventually experienced post-treatment relapses, which were either immediate (trastuzumab-containing triplet) or delayed (mAb33-containing triplet).

To resolve the molecular bases of the cooperative drug effects, we re-performed the PDX trial, but mice were sacrificed after seven days of treatment and tumors were subjected to immunofluorescence and immunoblotting. Unlike the trastuzumab-containing triplet, which strongly downregulated HER2, the other triplet exerted no effect on the abundance of HER2, but partly reduced HER3 (Figure S4A). Nevertheless, both triplets completely blocked phosphorylation of EGFR, HER2, and HER3, as well as reduced activation of AKT and ERK, and downregulated both AXL and another receptor, which is not presented, VEGFR2 (vascular endothelial growth factor 2). Interestingly, the trastuzumab-containing triplet more strongly downregulated MET and only partly inhibited ERK. Presumably, some differences might be attributed to technical issues relevant to tissue processing, inter-animal variation, and premature tumor harvest. Nevertheless, consistent with the overall similarity between the effects of the two triplets, thin sections prepared from each tumor and probed for a cell proliferation marker, KI67, indicated complete blockade of cell division (Figure S4B), which was attributable to the effect of osimertinib rather than the antibodies. Next, we stained additional thin sections from the same tumors with hematoxylin and eosin (Figure S5). We noted multiple mitotic figures (arrows) in the control group, as well as in the groups treated with the pairs of mAbs. In contrast, no mitotic figures were observed in the osimertinib-treated group and in both triple combination groups. Notably, these groups displayed more prominent papillary structures, which looked more fibrotic in comparison to the control group.

In conclusion, despite high aggressiveness of the metastasis-derived PDX, blocking either HER3 or HER2 markedly improved the outcome of animal treatments with osimertinib: Unlike the rapid relapses we observed while treating mice with osimertinib alone, both drug triplets regressed pre-formed tumors and prevented relapses, as long as treatment continued. Moreover, in comparison to other scenarios, the mAb33-containing triplet best delayed relapses post treatment.

### 2.7. Tumors Relapsing Post-Treatment with the Triplets Remain Sensitive to Renewed Triplet Applications

Because mice treated with a TKI plus a pair of mAbs displayed complete, or nearly complete, tumor inhibition, but all tumors relapsed after treatment cessation, we asked if the residual diseases that seeded regrowth of the PDXJ2 model retained sensitivity to the original triplet. To this end, relapsing tumors were re-treated as before (vertical arrows in the bottom panels of Figure 7A). The tumor growth curves presented in Figure 7B indicate that in all 10 mice we tested, from both groups, tumors were rapidly inhibited, indicating that no acquisition of resistance to the triplets took place. Hence, despite recurrence post treatment, the residual disease remains sensitive to re-treatment, indicating that no resistance emerged during the drug holiday.

In summary, previous studies indicated that blocking EGFR alone in lung cancer cannot prevent disease recurrence, because bypass routes inevitably instigate emergence of drug resistance. Our study demonstrates that blocking bypass routes mediated by either HER3 or HER2, concurrently with a dual blockade of EGFR, irreversibly inhibits a cell line xenograft, or confers long-term (but reversible) inhibitory effects in patient-derived models. These observations are novel because unlike HER2, a well-established target, inhibiting HER3 has remained an open issue. Employing our neutralizing antibody, we showed that the anti-HER3 mAb can block drug-induced up-regulation of HER3, as well as shedding of soluble HER3. Moreover, in combination with EGFR inhibitors, the antibody downregulates other resistance-associated receptors, such as AXL and MET, thereby blocks AKT signaling and promotes apoptosis. Because our study simulated in animals the situation of patients with NSCLC, who are currently developing resistance to third-generation TKIs and remain with no viable treatment options, the lessons learnt in the course of this study warrant safety tests, which might lead to advanced clinical trials.

## 3. Discussion

Although EGFR is mutated in a large fraction of NSCLC, and it acts as an established tumor driver, under pharmacological stress this driver seems to be replaced by an alliance comprising, in addition to EGFR, also HER2 and HER3. One exemplification of this model is the unique ability of a combination comprising osimertinib and the respective three mAbs to strongly inhibit TKI-resistant cell line xenografts [28]. Herein we used the more heterogeneous PDX models, and demonstrated that blocking either pair of receptors, EGFR + HER3 or EGFR + HER2, confers inhibition of recurrence. The alliance of the three RTKs is likely mediated by means of heterodimer formation; EGFR forms heterodimers with both HER2 and HER3, and, in addition, HER2 and HER3 can form heterodimers [39]. Moreover, previous studies detected heterodimer formation between EGFR and MET, IGF1R, and AXL [40,41,42]. Likewise, it has been shown that compensatory loops involving alternative receptors, and mediated by EHF, an ETS family transcription factor, are activated when EGFR is inhibited [43].

In addition to HER3′s propensity to form heterodimers, this receptor can launch potent bypass pathways through AKT [44]. Hence, it is not surprising that HER3 initiates emergence of secondary resistance. Examples include HER3 up-regulation following long-term exposure to trastuzumab [45] or TKI-induced inhibition of EGFR and HER2 [22,26]. Importantly, when co-administered with a TKI, the cocktail of two mAbs we applied (i.e., mAb33 plus cetuximab) induced downregulation of additional RTKs, including AXL and MET. Although it is still unknown why these non-HER proteins do not elevate following treatment with the triple combination of drugs, our observations support the following model: In response to stress, lung cells stimulate a compensatory mechanism that transcriptionally elevates HER3. This receptor forms heterodimers with several RTKs and enhances cell survival by means of activating AKT. Although treatments making use of a neutralizing anti-HER3 mAb do not block transcription-mediated up-regulation, they enhance degradation of HER3. Presumably, by forming heterodimers, HER3 stabilizes other RTKs, such that HER3 degradation inactivates several RTKs.

Although both drug triplets we applied inhibited NSCLC models, the anti-HER3 triplet displayed consistent superiority. One potential reason relates to the different spectra of blocked growth factors: Unlike the HER2-blocking triplet, the one blocking HER3 is expected to inhibit both neuregulins and all EGFR ligands. In line with this scheme, analyses of sera reported that patients who present high serum neuregulins better respond to an anti-HER3 antibody [46]. Similarly, important, the anti-HER3 mAb, unlike trastuzumab, can inhibit up-regulation of HER3 in response to osimertinib. Yet another functional difference between HER3 and HER2 entails ectodomain cleavage and shedding. Herein we report that HER3 cleavage is mediated by ADAM10 and this event is enhanced by osimertinib. Cleavage of HER2 is mediated by both matriptase [47] and ADAM 10 [48], and generates soluble molecules, as well as a constitutively active truncated intracellular receptor (p95HER2) [49]. In this context, it is worthwhile referring to the ability of MEK inhibitors to decrease shedding of MET, as well as AXL, thus increasing surface RTK levels and mitogenic signaling [50].

Unlike HER3, HER2 can act as a potent tumor driver, for example in breast and lung cancer [51]. In contrast, HER3 rarely acts as a cancer driver; only very few oncogenic mutations were identified in gastrointestinal cancers [52]. In accordance, six anti-HER2 drugs are already in common use against HER2-positive tumors, but no anti-HER3 drug has so far been approved. Nevertheless, many studies reported that HER3 plays important roles in the progression of a variety of tumor types, such as castration-resistant prostate cancer [53]. Additional examples, along with the wide occurrence of adaptive up-regulation of HER3 in response to stress, predict that anti-HER3 drugs might be efficacious, especially in combination with other drugs. In similarity to the study reported herein, a recent report found that triple blockade of HER2 and HER3 using two anti-HER2 antibodies and a third, an anti-HER3 mAb, could overcome resistance to trastuzumab [54], and yet another study reported that an anti-HER3 antibody can sensitize refractory NSCLC to erlotinib [55]. Hence, it is tempting to predict that HER3-targeting agents will be included in future treatment protocols.

## 4. Materials and Methods

### 4.1. Cell Cultures and Reagents

H1975 and PC9ER cells were obtained from ATCC and from Julian Downward (F. Crick Institute, London, UK), respectively. Cells were authenticated as per ATCC standards. Osimertinib was a gift from Astrazeneca (Cambridge, UK). Cetuximab and trastuzumab were obtained from Merck (Darmstadt, Germany) and Roche (Basel, Switzerland), respectively. The murine antibody mAb33 was generated in our lab [32]. The anti-GAPDH antibody was obtained from Millipore (Burlington, MA, USA) and the anti-human β1-tubulin from R&D Systems (Minneapolis, MN, USA). All other antibodies were obtained from Cell Signaling Technology (Danvers, MA, USA).

### 4.2. Thymidine Incorporation Assay

Cells were plated onto 24-well plates and after 12 h they were pulsed with 3H-thymidine (1 µCi) in fresh, serum-free media. The reaction was terminated 48 h later by adding ice-cold trichloroacetic acid (TCA; 5%, 5 min on ice), followed by successive washing with 1N NaOH and 1N HCL (1 mL per well, 10 min at 37 °C). Samples were mixed with scintillation fluid and radioactivity was determined.

### 4.3. Migration and Invasion Assays

PC9ER or H1975 cells, which were pretreated with the indicated drugs, were trypsinized, washed, and resuspended in medium at a density of 4 × 10^5^ cells/mL (for migration) or 8 × 10^5^ cells/mL (for invasion). This cell suspension (0.1 mL) was added to the upper chamber of a Transwell tray (Corning, migration) or to BioCoat Matrigel Invasion Chambers (Corning, NY, USA). The lower chambers were filled with 0.5 mL of medium. Following incubation for 20 h (migration) or 22 h (invasion), cells were fixed in 4% formaldehyde and stained at room temperature for 15 min with crystal violet (5%). The filters were then rinsed thoroughly in distilled water. Non-migrating cells were removed from the upper side of the filter using a wet cotton swab. To quantify cell motility, cells that had migrated or invaded to the bottom surface (migration) or bottom well (invasion) of the inserts were counted. A total of five evenly spaced fields of cells in each well were captured using a phase-contrast microscope and signals were quantified using ImageJ. Significance was assessed using one-way ANOVA, followed by Dunnett’s or Tukey’s Multiple Comparison Test.

### 4.4. Colony Outgrowth Assay

Cells were seeded in 12-well plates at a density of 1000 cells per well. Drugs were added 24 h later and incubated for 9–10 additional days. Media were refreshed once in 3 days. Thereafter, cells were fixed in formaldehyde (4%) and stained with crystal violet. Photos were taken using an EPSON PERFECTION 4870 Photo Scanner (Long Beach, CA, USA). Growth was quantified by dissolving cells in detergent solution (2%  SDS) and determining light absorbance (590 nm).

### 4.5. Immunoblotting Analyses

Cells were washed twice with phosphate buffer saline (PBS) before adding lysis buffer (50 mM Tris, pH 7.5, 10% glycerol, 150 mM NaCl, 1% Nonidet P-40, 1 mM EDTA, 0.5% sodium deoxycholate 0.1 mM Na_3_VO_4_ and a complete protease inhibitor cocktail). Tumor xenografts were processed using the gentleMACS™ Dissociator (Miltenyi Biotec, Bergisch Gladbach, Germany) in lysis buffer. Protein extracts were resolved using gel electrophoresis and transferred onto nitrocellulose membranes. After blocking, membranes were incubated overnight with the indicated primary antibodies, followed by incubation for 1 h with horseradish peroxidase–conjugated secondary antibodies (from Jackson ImmunoResearch Laboratories) and probed with ECL PlusWestern blotting Detection System (GE Healthcare Bio- Sciences, Chicago, IL, USA). Signals were detected using the ChemiDoc™ Imaging System (Bio-Rad) and images were acquired using the ImageLab Software, 6.0.1.

### 4.6. RNA Isolation and Real-Time PCR Analyses

PC9ER or H1975 cells were treated for 24 h with the indicated drugs and total RNA was extracted using the miRNeasy Mini Kit (QIAGEN, Hilden, Germany), according to the manufacturer’s instructions. The complementary cDNA obtained using the qScript cDNA Synthesis Kit (Quantabio, Beverly, MA, USA) was used as template for the real time quantitative PCR (qPCR) analyses. qPCR was performed using SYBR green (Applied Biosystem, Waltham, MA, USA) and specific primers. qPCR signals (Ct) were normalized to beta2-microglobulin (B2M).

### 4.7. Determination of Receptor Abundance on the Cell Surface

Cells were seeded in 6-well plates (5 × 10^5^ per well). On the next day, media were replaced with media containing 1% serum and the cells were treated with drugs for additional 24 h. Thereafter, cells were washed in acidic buffer (glycine-HCl 100 mM, pH 3.0) and detached from the plate using trypsin. Finally, cells were washed twice in saline containing albumin (1% *w*/*v*) and incubated for 30 min at 4 °C using antibodies to EGFR (clone AY13), HER2 (clone 24D2), and HER3 (clone 1B4C3), which were conjugated respectively to the following fluorophores: Alexa Fluor 488, allophycocyanin, and phycoerythrin (BioLegend Inc, San Diego, CA, USA). Fluorescence intensity was measured using the BD LSR II cytometer (BD Biosciences, San Jose, CA, USA) and analyzed by BD FACS Diva software (BD Biosciences).

### 4.8. ImageStream Analysis

Cells were treated with the indicated drugs and 24 h later they were washed in acidic buffer (100 mM glycine-HCl, pH 3.0). Permeabilization was carried out by adding 0.05% Triton X-100. Thereafter, cells were washed twice followed by blocking with albumin (3%). Next, cells were stained with the corresponding primary conjugated antibodies. Samples were analyzed using the ImageStream Mk II Imaging Flow Cytometer (EMD Amnis, Seattle, WA, USA). Data were assessed using the GraphPad software, version 8.4.3.

### 4.9. Immunofluorescence

Tissue cultures: PC9ER cells were grown on autoclaved coverslips in 6-well plates. Following treatments, cells were washed in saline containing Tween 20 (0.1%; *w*/*v*; PBS-T), followed by incubation with glycine buffer (100 mM, pH 3; 5 min). Thereafter, cells were fixed in formaldehyde (4%; overnight at 4 °C). Next, cells were washed and permeabilized (in saline with 0.1% Triton X-100). Blocking was carried out for 30 min using 2% fetal bovine serum (FBS), followed by incubation with a primary antibody in PBS-T containing FBS (1%; at 4 °C, overnight). Thereafter, cells were washed thrice followed by staining with a FITC-conjugated secondary antibody and DAPI (45 min in dark). Images were captured using a Zeiss confocal microscope (63× magnification) and processed using the Zeiss ZEN2011 software (Blue Edition).

Tumor specimens from mice: Formalin-fixed paraffin-embedded (FFPE) tissue specimens were deparaffinized in xylene and rehydrated in graded ethanol. Antigen retrieval was performed using a citric acid solution (pH 9.0; in microwave, 10 min). After three washes in saline, the slides were blocked in buffer containing 20% normal horse serum, followed by treatment with an avidin/biotin blocking solution (15 min) and an overnight incubation with the corresponding primary antibody. Slides were incubated for 12 h at room temperature followed by incubation for 24 h at 4 ºC. Sections were washed and incubated with a biotinylated anti-rabbit secondary antibody, for 90 min at RT, followed by Cy3-conjugated streptavidin. Thereafter, the sections were washed, and nuclei were stained with DAPI. Finally, each slide was treated with mounting medium (Aqua Poly/Mount, Polysciences, Warrington, PA, USA) and examined on the next day using a fluorescence microscope (Eclipse Ni-U, Nikon, Tokyo, Japan) equipped with Plan Fluor objectives (6x) connected to a monochrome camera (DS-Qi1, Nikon, Tokyo, Japan). Positive cells were counted using the Image Pro Plus software (version 4.1).

### 4.10. Animal Experiments

All animal studies were approved by the Weizmann Institute’s Animal Care and Use Committee (IACUC) and the Institute’s Review Board (IRB). Athymic 6-week-old female CD-1 nu/nu mice were injected subcutaneously with PC9ER cells (3–4 × 10^6^ per mouse). Two lung PDX models, TM00204 (PDXJ1) and TM00219 (PDXJ2), were obtained from The Jackson Laboratory and expanded in NSG mice. A third model, TP103, was implanted in CD-1 nu/nu mice. Following euthanasia, tumors were removed from donor mice and cut into small fragments. A small pouch was made in the lower back of male or female mice, 5-6 weeks old, and one fragment was later inserted into the pouch. Mice were labelled with RF identification chips (from Trovan, Melton, UK). Antibodies were injected intraperitoneally at 0.2 mg (total) per mouse per injection, twice weekly. Daily administration of osimertinib used oral gavage. Tumor width and length were measured twice a week using a caliper, and tumor volume (V) was calculated using the formula 3.14 × (shortest diameter) × (longest diameter)^2^ × 1/6. Mice were euthanized when tumors reached 1500 mm^3^ (PC9ER xenografts) or 1200 mm^3^ (PDX models).

### 4.11. Statistical Analysis

Significance was assessed using one- or two-way ANOVA followed by Tukey’s, Sidak’s, or Dunnett’s multiple comparison test (**** *p* < 0.0001, *** *p* < 0.001, ** *p* < 0.01, * *p* < 0.05). All experiments were repeated thrice, at least in triplicates, unless otherwise indicated.

## 5. Conclusions

Our study concludes that despite the fact that HER3 is nearly devoid of kinase activity, it can still serve as an effective drug target in the context of acquired resistance to EGFR-specific kinase inhibitors. This has been demonstrated herein using in vitro and animal models, which employed cell line xenografts, as well as patient-derived lung tumors. The neutralizing anti-HER3 antibody we generated in our laboratory can clear HER3 from the cell surface, as well as reduce HER3 cleavage by metalloproteinases. In line with these activities, when combined with a third-generation EGFR inhibitor, osimertinib, and cetuximab, a clinically approved anti-EGFR antibody, the anti-HER3 antibody prevented emergence of resistance to osimertinib. We infer that the combination of three drugs acts by means of preventing osimertinib-induced up-regulation of HER3, suppressing resistance-conferring receptors, and inducing BIM-mediated apoptosis. We conclude that the new drug combination offers pharmacological opportunities.

## Supplementary Materials

The following are available online at https://www.mdpi.com/2072-6694/12/9/2394/s1, Figure S1: Transcripts encoding HER3 are upregulated after treatment of lung cancer cells with osimertinib, but the protein undergoes downregulation after treatment with an anti-HER3 antibody; Figure S2: Unlike osimertinib, the mAb33 cannot up-regulate sHER3, but in combination with osimertinib and cetuximab the antibody downregulates HER3 and inhibits activation of AKT; Figure S3: Combining a TKI and antibodies neutralizing EGFR and HER3 strongly decreases proliferation, migration, invasion and clonogenicity of H1975 cells (L858R EGFR); Figure S4: A patient-derived xenograft treated with combinations of osimertinib and a pair of mAbs displays downregulation of multiple signaling pathways, along with reduced cell proliferation; Figure S5: Triple drug combinations comprising a TKI and two antibodies increase papillary structures and decrease mitotic figures in a PDX model of NSCLC; Figure S6: Effects of animal treatment on body weight.
