# Supplementary material for: Targeting HER3, a Catalytically Defective Receptor Tyrosine Kinase, Prevents Resistance of Lung Cancer to a Third-Generation EGFR Kinase Inhibitor
Source: Cancers (Basel). 2020 Aug 24;12(9):2394. doi: 10.3390/cancers12092394 (PMC7563838; doi:10.3390/cancers12092394)
Supplement: Supplementary file 1 [file cancers-12-02394-s001.pdf]

## Supplementary Material

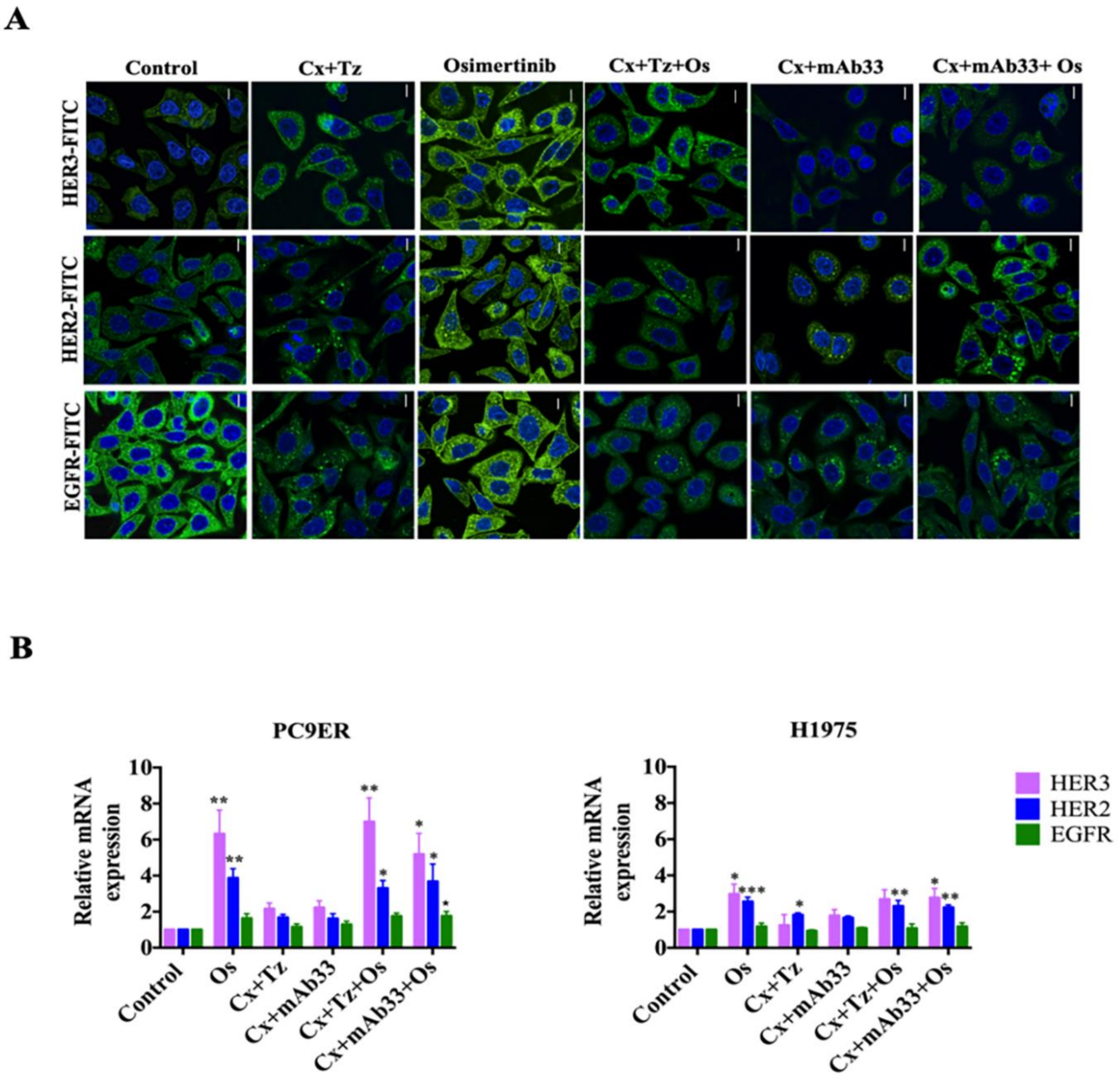


**Figure S1.** Transcripts encoding HER3 are upregulated after treatment of lung cancer cells with osimertinib, but the protein undergoes downregulation after treatment with an anti-HER3 antibody. (**A**) PC9ER cells were seeded on cover slips and allowed to grow for 24 hours. Thereafter, cells were treated with saline (*Control*), 2XmAbs (cetuximab plus mAb33 or cetuximab (Cx) plus trastuzumab (Tz); 10 μg/mL, each), osimertinib (Os, 40 nM), or with the indicated drug combinations (2XmAbs plus osimertinib). Samples treated with antibodies were incubated with an acidic solution for 5 minutes. Cells were later fixed in formaldehyde (4%) and incubated overnight with specific primary antibodies, followed by a secondary, FITC-conjugated antibody (45 minutes at room temperature). DAPI staining (blue) indicates locations of nuclei. Images were captured using a confocal microscope (63× magnification). Bars (white, vertical), 10 μm. (**B**) PC9ER or H1975 cells were treated for 24 hours as in A. RNA was extracted and RT-qPCR was performed to analyze transcript levels of *EGFR*, *HER2* and *HER3*. The experiment was repeated three times, in triplicates. Significance was assessed using one-way ANOVA followed by Dunnett's multiple comparison test. * $p < 0.05$, ** $p < 0.01$, *** $p < 0.001$.

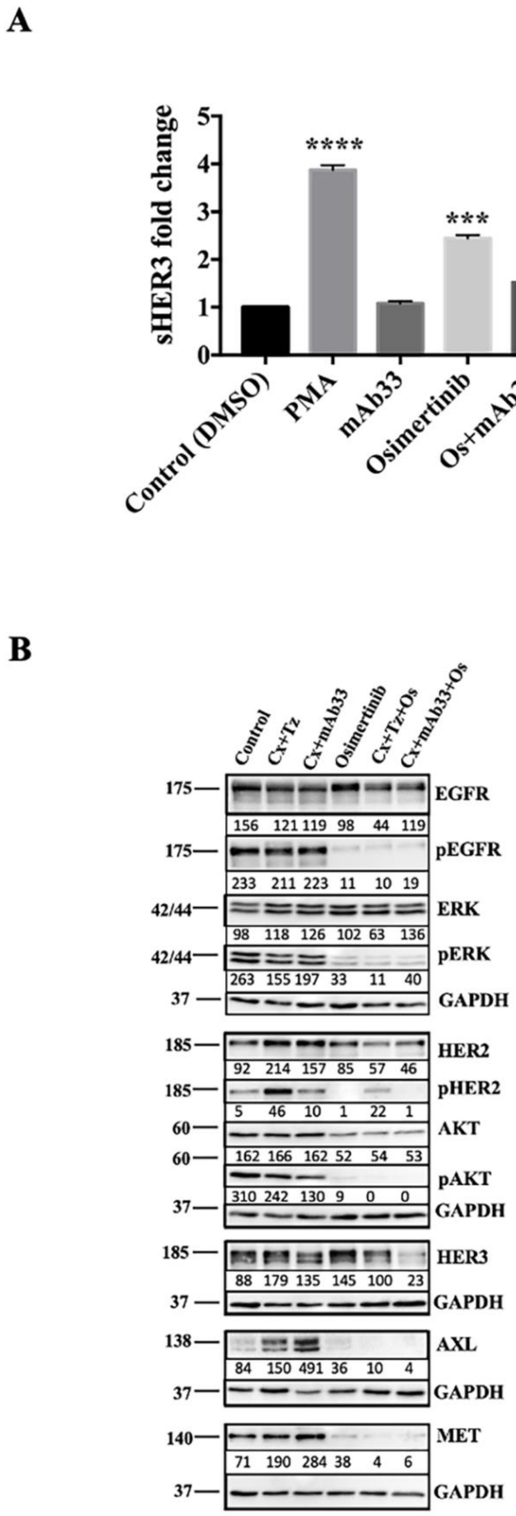


**Figure S2.** Unlike osimertinib, the mAb33 cannot up-regulate sHER3, but in combination with osimertinib and cetuximab the antibody downregulates HER3 and inhibits activation of AKT. (**A**) 96-well trays were incubated overnight with mAb252 (2 μg/mL), which recognizes an epitope of HER3, which is distinct from the one recognized by

mAb33. On the next day, wells were blocked and later incubated for 2 hours with medium conditioned by PC9ER cells overexpressing a double peptide-tagged HER3. Prior to media harvesting, cells were pre-treated with DMSO, phorbol myristate acetate (PMA, 100 nM), osimertinib (40 nM), mAb33 (20 μg/ml) or a combination of osimertinib and mAb33. Thereafter, all wells were incubated for 2 hours with an anti-HA antibody, and later with an anti-rabbit mAb conjugated to HRP. This was followed, 30-minutes later, by an incubation with ABTS (2,2′-azino-bis(3-ethylbenzothiazoline-6-sulfonic acid)). Signals were determined using an ELISA reader (set at 420 nm). Values represent averages of duplicates. Significance was assessed using one-way ANOVA followed by Dunnett's multiple comparison test. *** $p < 0.001$, **** $p < 0.0001$. (**B**) PC9ER cells were treated for 48 hours with osimertinib (40 nM), the indicated pairs of antibodies (10 μg/ml each), and the respective drug combinations. Cell extracts were blotted and probed for the indicated proteins. GAPDH was used as a loading control protein. Numbers below each lane refer to densitometrically determined ratios between band intensity and GAPDH level.

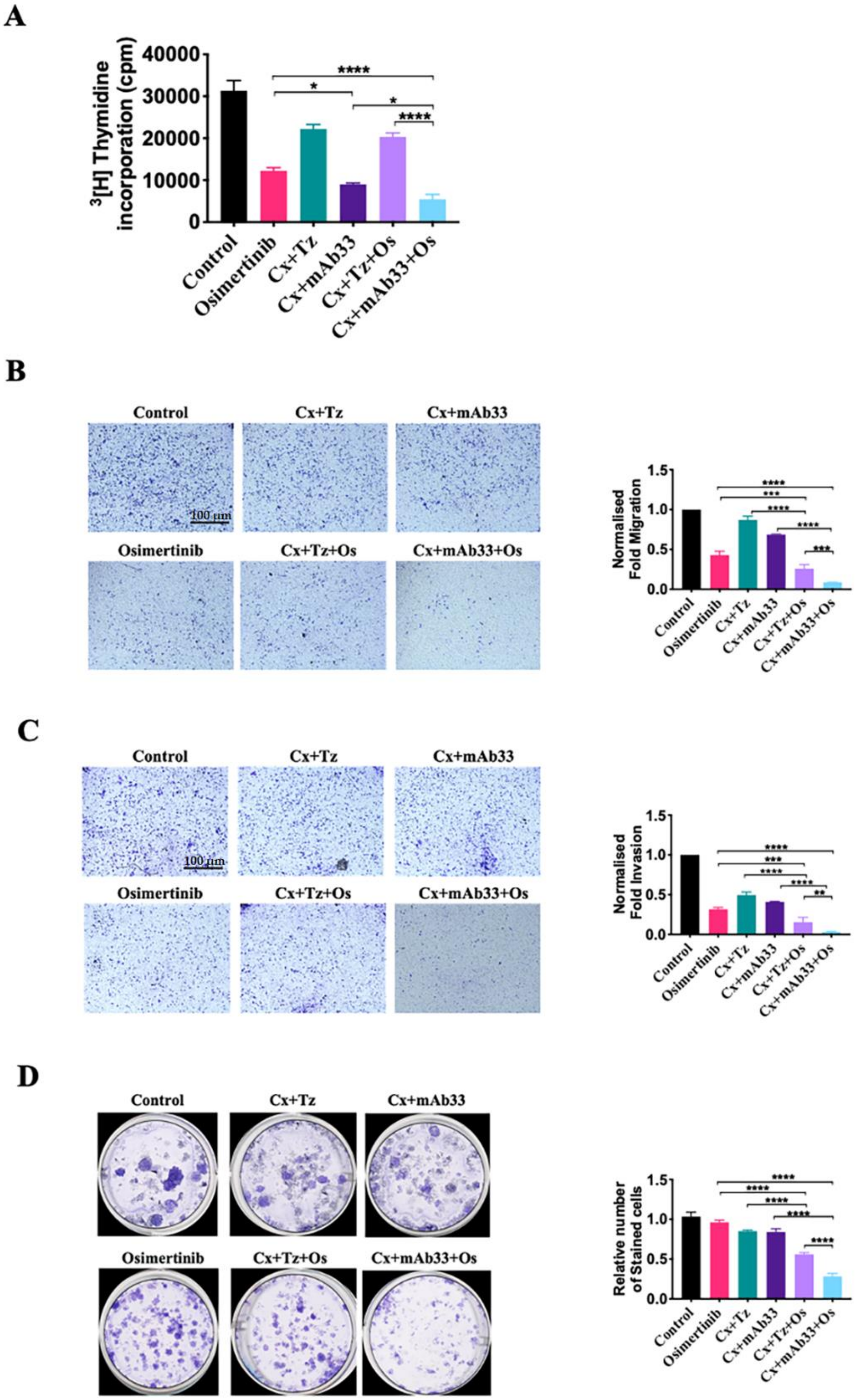


**Figure S3.** Combining a TKI and antibodies neutralizing EGFR and HER3 strongly decreases proliferation, migration, invasion and clonogenicity of H1975 cells (L858R EGFR). (**A**) Radioactive thymidine incorporation assays were performed with H1975 cells pre-plated onto 24-well plates at a density of 2 × $10^4$ cells/well. After 12 hours the medium was replaced with fresh serum-free medium containing different treatments, as follows: vehicle control, a pair of mAbs, either cetuximab (Cx) + trastuzumab (Tz) or cetuximab + mAb33 (each antibody at 10 µg/ml), osimertinib (40 nM), and

the indicated drug combinations, along with $^3$H-thymidine (1 μCi). Forty-eight hours later, radioactivity was determined in a scintillation counter. Significance was assessed using two-way ANOVA followed by Sidak's multiple comparison test. Values represent averages ± SEM. The experiment was repeated twice, in quadruplicates. * $p < 0.05$, **** $p < 0.0001$. (**B** and **C**) Cell migration and invasion assays were performed with H1975 cells pre-treated with the following agents for 48 hours: the indicated pairs of mAbs (10 μg/ml each), osimertinib (40 nM) and the respective combined treatments. Pretreated cells were seeded in triplicates onto Transwells or invasion wells. Migration and invasion were carried out for 20 and 22 hours, respectively, in complete medium. The panels show representative cell images and histograms, which present quantification of migrated cells relative to control. Significance was assessed using one-way ANOVA followed by Sidak's multiple comparison test. Values represent averages ± SEM. ** $p < 0.01$, *** $p < 0.001$, **** $p < 0.0001$ Bars, 100 μm. The above results are representative of three biological experiments, which were performed in triplicates. (**D**) Colony outgrowth assays were performed using crystal violet staining and quantification. H1975 cells ($1 \times 10^3$) were seeded in 12-well microplates. After 24 hours, the cells were exposed to the following drugs for 9–10 days: osimertinib (40 nM) or the indicated pair of mAbs (each at 10 μg/mL), either alone or with osimertinib. Later, the cells were fixed with formaldehyde (4%) and stained with crystal violet (0.5%). Photos of stained cells were captured using an EPSON scanner. Cell growth was quantified by dissolving crystal violet in SDS (2%). Absorbance was quantified at 590 nm. The experiment was repeated three times, in triplicates. Significance was assessed using one-way ANOVA with Tukey's multiple comparison test. **** $p < 0.0001$.

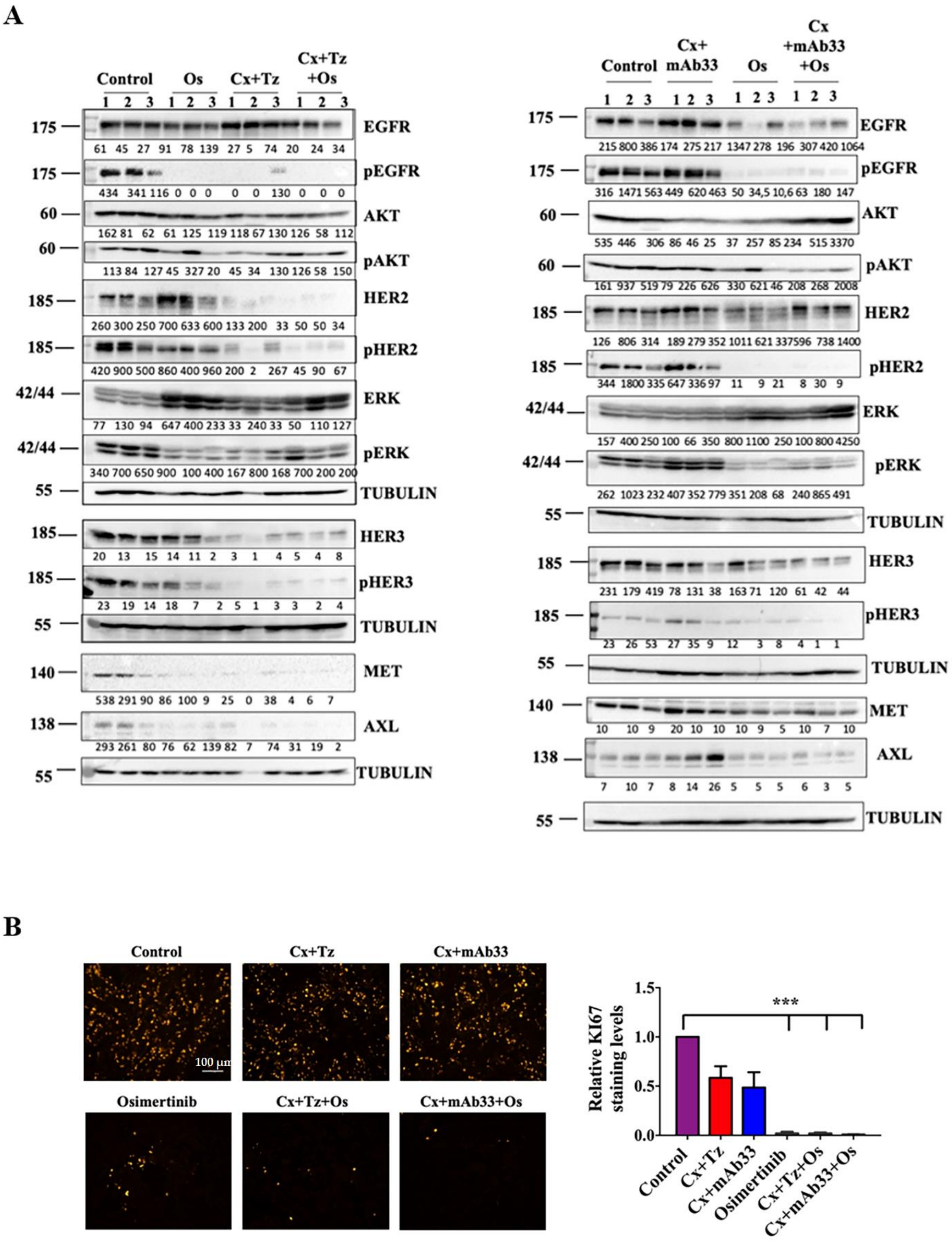


**Figure S4.** A patient-derived xenograft treated with combinations of osimertinib and a pair of mAbs displays downregulation of multiple signaling pathways, along with reduced cell proliferation. Mice harboring PDXJ2 tumors were treated daily with osimertinib (10 mg/kg/dose), the indicated pairs of antibodies (0.2 mg/mouse/injection) or with

a combination of the TKI and either pair of mAbs. The antibodies were administered intraperitoneally once every three days. Following one week of treatment, three tumors of each group were collected for immunoblot analyses that used the indicated antibodies (**A**), or for fixation in formalin and embedding in paraffin (**B**). Numbers in A refer to quantification of band intensity, relative to tubulin. Sections (2 μm) in B were obtained from each tissue block and analyzed by means of immunofluorescence, which utilized a rabbit anti-KI67 antibody. KI67-positive cells were counted using the Image Pro Plus software. Shown are the resulting tissue sections along with a histogram presenting means ± SEM of two experiments. Significance was assessed using one-way ANOVA with Dunnett's multiple comparison test. *** $p < 0.001$. Note that the differences among the three treatments that included osimertinib achieved no statistical significance. Scale bar, 100 μm.

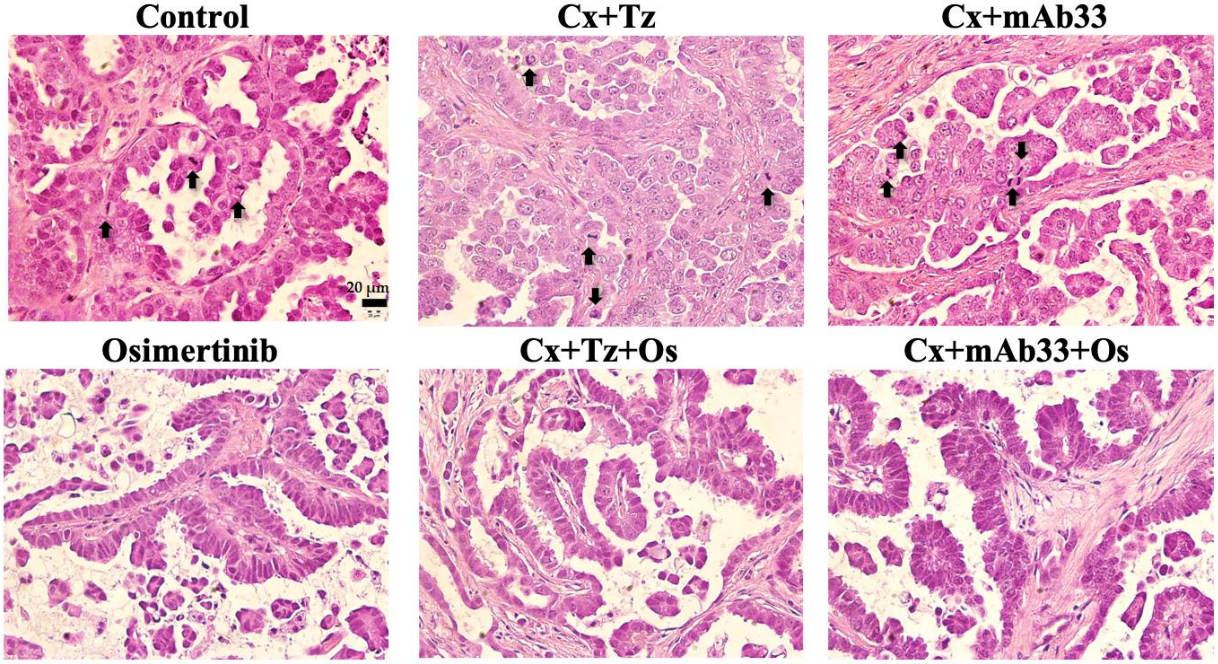


**Figure S5.** Triple drug combinations comprising a TKI and two antibodies increase papillary structures and decrease mitotic figures in a PDX model of NSCLC. Shown are photomicrographs of hematoxylin and eosin (H&E) stained sections of tumors pre-implanted in mice. The original tumors were derived from a metastatic lesion of a patient with NSCLC expressing an EGFR with a deletion in exon 19 and the T790M mutation (PDXJ2). Mice were treated with drugs for one week. The following treatments were used: daily oral delivery of osimertinib (10 mg/kg/dose) and two treatments with pairs of antibodies (Cx + Tz or Cx + mAb33; 0.2 mg/mouse/injection), which were administered intraperitoneally. Two additional groups of mice were treated with combinations of two mAbs and osimertinib, as indicated. Seven days after treatment onset, three tumors of each group were harvested, formalin fixed and paraffin embedded. Photos are representative sections from three independent experiments. Five or more fields were analyzed per treatment. Note that mitotic figures are marked using arrows. Bar, 20 μm.

**A**

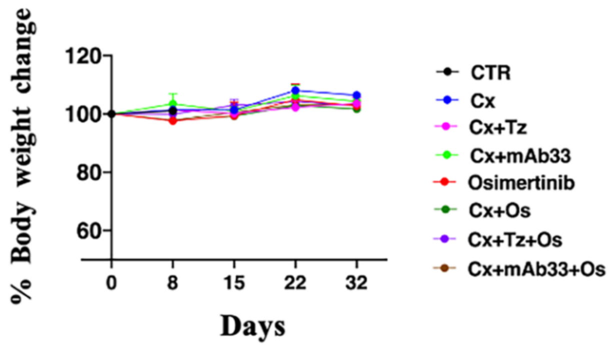


**B**

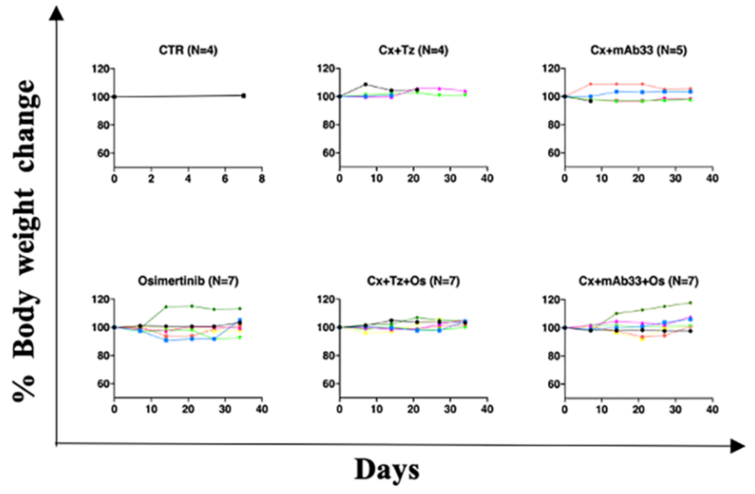


**C**

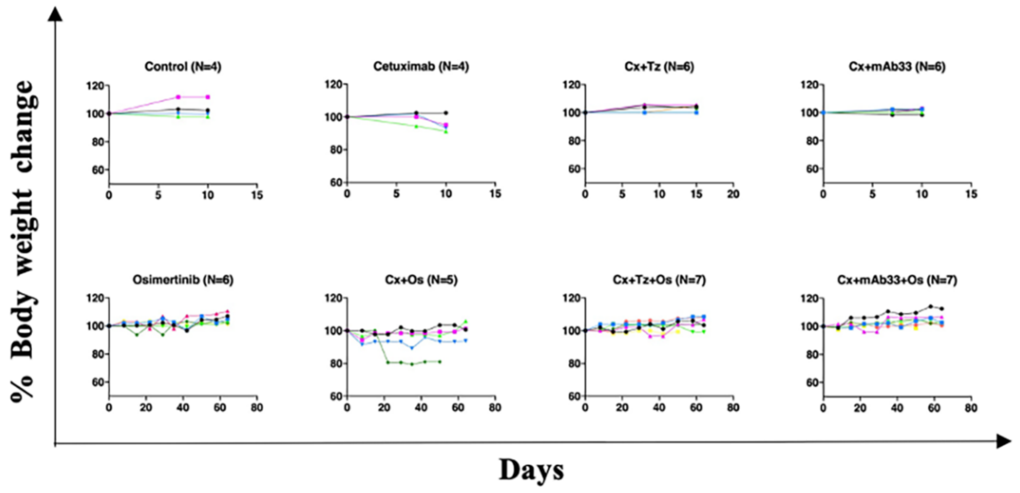


**Figure S6.** Effects of animal treatments on body weight. (**A**) CD1 nu/nu mice were injected with PC9ER cells ($3 \times 10^6$ cells per mouse) and mice harbouring palpable tumors were divided into the listed eight different

treatment groups, as indicated in the legend to Figure 5A. Treatments continued until day 21. Tumor volume was monitored twice a week and body weight was measured once per week. Shown are the averages of body weights (± SEM) for each group of mice. The following drug doses were used: osimertinib (5 mg/kg/day) and mAbs (0.2 mg/mouse/injection when delivered alone or 0.1 mg per animal, per injection, when combined with another mAb). (**B**) The PDX model TM00204 (PDXJ1) was engrafted in NSG mice, which were later treated as follows (see Figure 6A): control (*CTR*), osimertinib (daily oral gavage, 10 mg/kg), antibody pairs (each mAb at 0.1 mg/injection), either cetuximab (Cx) plus trastuzumab (Tz; intraperitoneal delivery, twice per week), or cetuximab plus mAb33. Two additional groups of animals were similarly treated with osimertinib in combination with the indicated pairs of mAbs. The number (N) of mice per group is indicated. Body weights of individual animals are reported for each treatment. (**C**) The lung PDX model TM00219 (PDXJ2) was engrafted in NSG mice, which were later divided into the indicated 8 groups (see Figure 7A). The doses of drugs were as follows: osimertinib, 10 mg/kg/mouse); single mAbs, 0.2 mg per injection; pairs of antibodies, each at 0.1 mg per injection. Body weights were measured once per week and presented for each mouse. Note that treatment length varied.
